# Cutaneous Manifestations of Spotted Fever Rickettsial Infections in the Central Province of Sri Lanka: A Descriptive Study

**DOI:** 10.1371/journal.pntd.0003179

**Published:** 2014-09-18

**Authors:** Kosala Weerakoon, Senanayake A. M. Kularatne, Jayanthe Rajapakse, Sanjaya Adikari, Roshitha Waduge

**Affiliations:** 1 Department of Parasitology, Faculty of Medicine and Allied Sciences, Rajarata University of Sri Lanka, Saliyapura, Sri Lanka; 2 Department of Medicine, Faculty of Medicine, University of Peradeniya, Peradeniya, Sri Lanka; 3 Department of Veterinary Pathobiology, Faculty of Veterinary Medicine and Animal Science, University of Peradeniya, Peradeniya, Sri Lanka; 4 Department of Anatomy, Faculty of Medicine, University of Peradeniya, Peradeniya, Sri Lanka; 5 Department of Pathology, Faculty of Medicine, University of Peradeniya, Peradeniya, Sri Lanka; University of California, San Diego School of Medicine, United States of America

## Abstract

**Background:**

Characteristic skin lesions play a key role in clinical diagnosis of spotted fever group rickettsioses and this study describes these cutaneous manifestations along with basic histological features.

**Methods and Findings:**

Study was conducted at Medical Unit, Teaching Hospital, Peradeniya, from November 2009 to October 2011, where a prospective data base of all rickettsial infections is maintained. Confirmation of diagnosis was made when IgM and IgG immunofluorescent antibody titre of 1/32 and >1/256 respectively. Of the 210 clinical cases, 134 had cutoff antibody titers for *Rickettsia conorii* antigen for confirmation. All these 134 patients had fever and skin rash, and of them 132(98%) had discrete maculopapular rash while eight (6%) had fern leaf type skin necrosis. Eight patients (6%) had healed tick bite marks. Average size of a skin lesion was 5 mm and rash involved 52% of body surface, distributed mainly in limbs and back of the chest. Generally the facial and leg skin was slightly oedematous particularly in old aged patients. Sixteen patients (12%) had pain and swelling of ankle joints where swelling extended to feet and leg. Biopsies from skin rash of six patients showed evidence of cutaneous vasculitis and of them, 247 bp region of the 17-kDa spotted fever group specific protein antigen was amplified using PCR.

**Conclusions:**

A discrete maculopapular rash and occasional variations such as fern leaf shape necrosis and arthritis are found in spotted fever group. Histology found vasculitis as the pathology of these lesions.

## Author Summary

Rickettsial organisms infect humans causing a wider array of clinical features and have re-emerged in Sri Lanka where three known disease entities; spotted fever group, murine typhus and scrub typhus do exist. These diseases cause clinical illnesses varying from mild febrile illness to severe multiple organ involvement even leading to fatal outcomes when there is a delay in diagnosis. Occasionally, clinical features could be nonspecific or atypical. Nevertheless, detection of skin lesions mostly facilitates the clinical diagnosis. Hence, clinicians need to be familiar with common as well as uncommon variations of skin manifestations. Being a treatable infection, early diagnosis is important and is heavily based on clinical features in settings where laboratory diagnostics are unavailable; at the same time delaying of treatment could lead to high morbidity and mortality. We identified some important variations of skin lesions associated with spotted fever group rickettsial infections which include fern leaf type skin necrosis mainly involving superficial skin with blackish discoloration which dries up with time and peels off. In addition, mild cutaneous oedema is seen over the face and ankles especially in older patients. Acute arthritis involving ankle joints were common manifestations which together with typical skin lesions facilitate the clinical diagnosis.

## Introduction

Rickettsiae are a group of alpha-proteobacteria found as an obligatory intracellular parasite of eukaryotic cells [1]. Rickettsia cause human infections giving rise to a wider array of clinical features. Rickettsial infections have re-emerged in Sri Lanka where three known disease entities namely spotted fever group (SFG), murine typhus and scrub typhus are being reported from different parts of the island [2]–[4]. Disease spectrum varies mainly depending on the rickettsial species that causes the disease, for example the Rocky Mountain spotted fever (RMSF) caused by *Rickettsia rickettsii* is known to be the most severe form of tick borne rickettsioses around the globe [1]. Clinical illness may vary from mild to severe with multiple organ involvement, sometimes leading to fatal outcomes [5], [6]. Generally, clinical features of the infection could be nonspecific or atypical. Nevertheless, the presence of cutanoeus lesions facilitates the clinical diagnosis of the infection. These include eschars, skin eruptions and rash with patchy necrosis [2], [4], [7]. Further, it is important to be familiar with the common cutaneous manifestations as well as uncommon variations of skin lesions. Being a treatable infection, early diagnosis is heavily based on clinical features in settings where laboratory diagnostics are not available and at the same time delaying of treatment could lead to high morbidity and mortality [8], [9]. Of the clinical features, cutaneous lesions play a major role that supports the diagnosis. However, these cutaneous lesions tend to have varying patterns influenced by many factors. Thus, clinicians need to get used to these variations to make a presumptive diagnosis of rickettsial infection. Moreover, identifying pathological changes of skin lesions are important as supportive tools in verifying the clinical diagnosis and also to understand the nature of the pathology caused by rickettsiae. The basic pathological changes have been described previously in other regions of the globe [10]. The aims of this study were to describe the morphology of cutaneous manifestations and their basic histological features of spotted fever rickettsial infections in Sri Lanka.

## Materials and Methods

### Setting

Patient recruitment and sample collection for the study were done in the Medical Unit, Teaching Hospital, Peradeniya from November 2009 to October 2011. This study was conducted according to the Declaration of Helsinki with approval from the Ethics review committee, Faculty of Medicine, University of Peradeniya, Sri Lanka. Informed written consent was obtained from all the adult patients and from guardians on behalf of the minors enrolled in the study.

### Study population and data collection

All patients admitted to the unit during the study period, with clinical features suggestive of rickettsial infection, were included in the study. Clinical case definition was based on the presence of fever for more than five days, associated skin rash and rapid defervescence with an anti-rickettsial antibiotic treatment [2], [4]. Patients were interviewed and examined, and followed up while in the hospital and after discharge. All clinical details were recorded on individual formatted data sheets, after obtaining the informed consent. Clinical details included the history, general examination and systemic examination findings. The details of the skin rash such as its distribution, size, and shape, presence of eschar and colour variations were recorded in a printed figure of the human body in the data sheet. The human body surface was divided into eight zones as, head and neck, anterior trunk, posterior trunk, arms and forearms, palms, thigh and gluteal regions, legs and soles. A score adopted from a locally devised method was used for each patient to indicate the extent of the skin rash [2]. A single score was given to each zone and the total extent of skin involvement was calculated as a percentage of skin finally.

In 2012, we saw two patients with extensive or unusual cutaneous manifestations who qualified for the diagnosis of rickettsial infections and included in this study as additional cases.

### Methods

Confirmation of diagnosis was made by positive serology using immunofluorescent antibody assays (IFA) and polymerase chain reaction (PCR). IFA test was carried out to detect antibodies against three groups of rickettsial antigens. Frozen antigen pellets of *Rickettsia conorii* (Strain Malish) of spotted fever group, *Orientia tsutsugamushi* (Strain Karp) of scrub typhus and *Rickettsia typhi* (Strain Wilmington) of murine typhus were used for slide preparation and the antigens were obtained from WHO Reference center for Rickettsial & Bartonella Associated diseases, CDC, Atlanta, USA. The final diagnosis of rickettsial infection was defined on the basis of their clinical criteria and the presence of specific IFA IgM and IgG seropositivity. Baseline cutoff titre value for IFA testing was 1/32, for both IgG and IgM. Samples with cutoff titre of IgG were further tested to obtain individual end point titres. Final serodiagnosis was based on the detection of IgM seropositivity and the IgG tire >1/256. The IgG titre above 1/256 has been used in published data in Sri Lanka where attempts have been made to validate this level as the standard cutoff value. Previous literature on serological values were used for the final disease confirmation despite some constraints [2], [11]–[13].

Cutaneous biopsy samples were obtained from six patients from areas with definitive maculopapular or vasculitic rash. A covered part of the body with the skin lesion was selected and the biopsy specimen was obtained under local anesthesia using a punch biopsy needle. Formalin fixed samples were processed and microscopic slides were prepared. Sections were stained with hematoxylin and eosin and were examined under microscope at magnifications of ×100, ×400, ×1000.

DNA was extracted from skin biopsy samples using QIAGEN spin column (Qiagen Sciences, Maryland 20874, USA) kit. Nested polymerase chain reaction (nPCR) assay was performed on extracted DNA to amplify the 17-kDa antigen gene. The primers used for primary and nested PCRs were R17-122, forward (5′- CAG AGT GCT ATG AAC AAA CAA GG-3′); R17-500, reverse (5′- CTT GCC ATT GCC CAT CAG GTT G -3′) and TZ 15, forward; (5′- TTC TCA ATT CGG TAA GGG C -3′) TZ 16, reverse (5′ - ATA TTG ACC AGT GCT ATT TC - 3′) respectively. DNA products were electrophoresed at 100 V. The eletrophoresed gel was observed under UV light (Gel documentation system, Vilber Lourmat, 77202 Marne la Vallee, France).

### Statistical analysis

Individual data points were stored in a computerized data base (Excel,Microsoft) and the basic descriptive analyses were done by means measures of central tendency. The data were analyzed by Minitab, version 14.0, Minitab Inc., USA.

## Results

A total of 210 patients qualified for the clinical diagnosis of rickettsial infections during the two year study period and their serum samples were tested for IgG and IgM antibodies for SFG, murine typhus group and scrub typhus groups of rickettsial infections. The average time gap between the onset of the illness and acute phase antibody testing was seven days. Ninety percent of the total (n = 188) showed positive titre above 1/32 of IgM and/or IgG to SFG. None of the patients had a positive IgG or IgM seroreativity for Typhus and Scrub Typhus groups alone. One patient showed a positive IgG seroreativity for both scrub typhus and SFG. Of the SFG, 164 (78%) had positive IgM seroreactivity and 188 (90%) had positive IgG seroreactivity. Of the IgG positive group, 144 patients had IgG titres >1/256. Moreover, both the IgM and IgG seropositivity was seen among 150 (71%) with 38 (18%) being positive only for IgG and 14 (7%) being positive only for IgM. Negative seroreactivity was observed in 22 (10%) patients of whom none of the serological tests were positive in acute sera. Of the total, 134 had high titers of IgM and IgG (IgG titre >1/256 and IgM titre >1/32) in the acute sera tested with IFA for *Rickettsia conorii* antigen (Table 1). This included 69 (52%) males and 65 (48%) females. Mean age of the group was 44 years (range, 12–84). Skin colour of the patients varied from fair to dark complexion with majority being dark skinned people.

**Table 1:** Details of IFA serology (IgG and IgM) in SFG cases.

IgG titire	IgM titre	Number of patients (%)^a^
≥1/256	>1/32	134 (64)^b^
>1/32 <1/256	>1/32	16 (8)
>1/32	<1/32	14 (7)
<1/32	>1/32	24 (11)
<1/32	<1/32	22 (10)^c^
**Total**		210 (100)

a Percentages were calculated, out of the total number of 210 patients.

b High titre positive cases presented in detail in this study.

c Group with negative seroreactivity.

All 134 patients with high antibody titers for SFG had fever and skin rash. Average duration of fever on admission was six days (range, 2–21days) and that of skin rash was two days (range, 1–7 days). Of this group, 119 (89%) had developed skin rash following the onset of fever and the average time gap between the appearance of these two symptoms was three days. The rest, 15 (11%) patients had developed fever and skin rash together.

**Figure 1:**
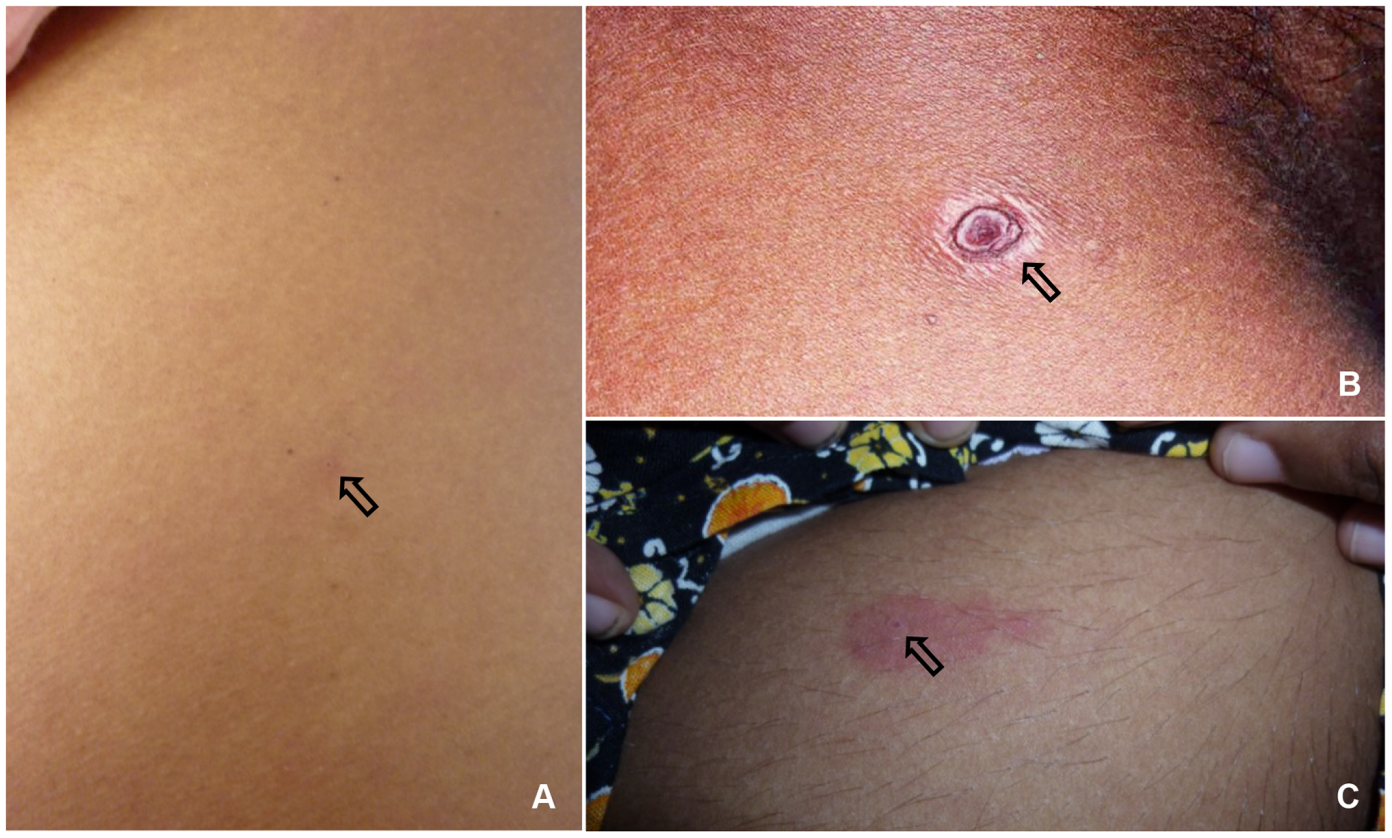
Tick bite marks found in patients, (A) a tick bite mark, ten days old, (B) a scarred tick bite mark, (C) a recent tick bite mark.

**Figure 2:**
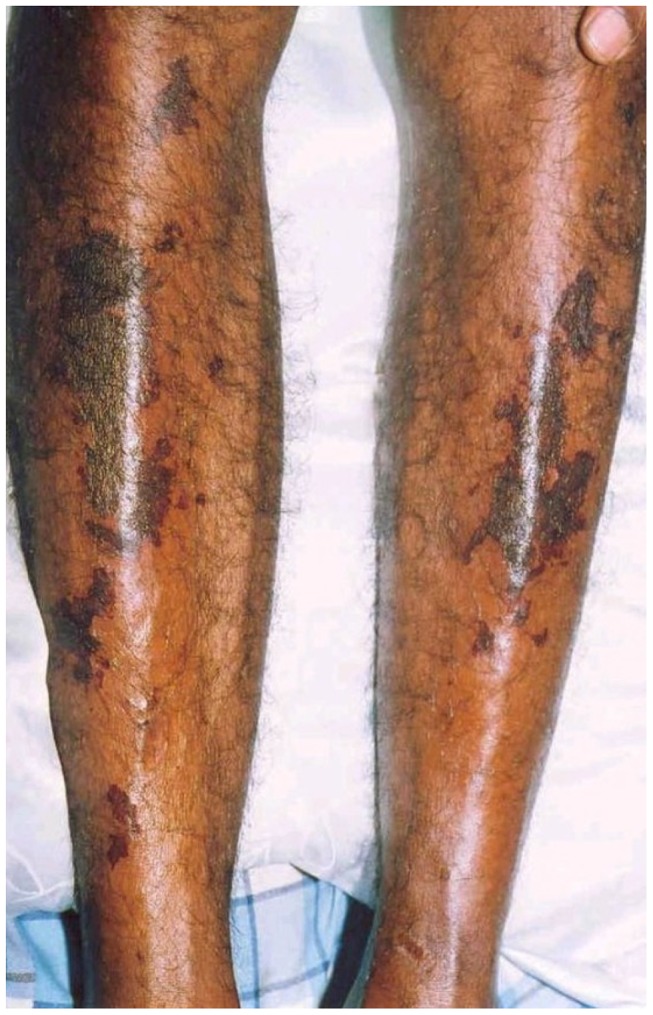
Fern leaf type skin necrosis in legs.

**Figure 3:**
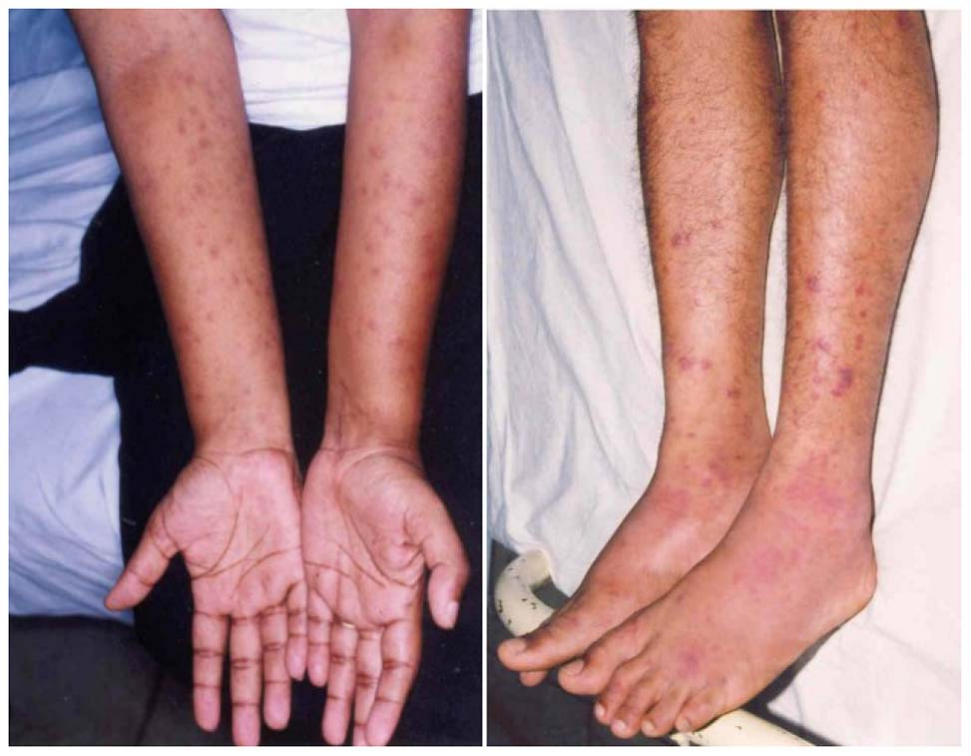
Erythematous or dusky red discrete skin rash in both upper and lower limbs involving hands and feet.

**Figure 4:**
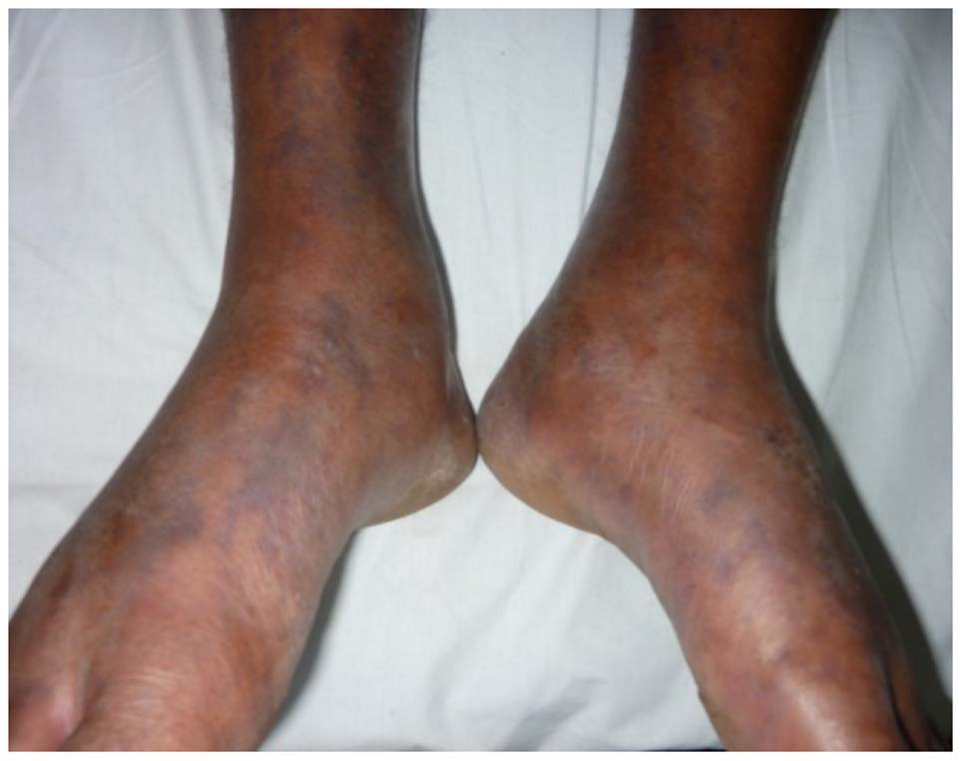
Acute arthritis of ankle joint and oedematous feet.

Of the cutaneous lesions, eight (6%) patients had healed tick bite marks apart from the skin rash (Figure 1). The skin rash was maculopapular in 132 (98.5%), macular in one patient and papular in another one. Eight patients (6%) of the group had fern leaf type skin necrosis (Figure 2) and the rest had erythematous rash (Figure 3), (Table 2). Mean age of the patients who had necrotic skin rash was 64 years. Appearance of the erythematous lesions varied depending on the innate skin colour of the subjects. Lesions were distinctively erythematous in fair skinned patients whereas those were more of dusky red in dark skinned patients. Furthermore, in some patients the rash was visible only if the skin was visualized from an angle in the daylight. Average size of a skin lesion was 5 mm and it ranged from 2–10 mm. The rash was always discrete with normal looking skin in between and the shape of the lesions varied. Commonly they were either ovular or round in shape. Necrotic rash had a fern leaf like pattern. These lesions took time to heal with black discoloration leaving pinkish base once peeled off. In general, the facial and leg skin of the patient seemed slightly oedematous particularly in old aged patients. Average extent of the skin involvement with the rash was 52% (range, 12.5–100%). Out of the eight body regions considered, majority of patients had the skin rash in their arms and forearms (n = 108, 81%) and legs (n = 90, 67%) while other areas involved were palms (74, 55%), soles (75, 56%), anterior trunk (61, 46%), thighs and gluteal region (53, 40%), head and neck (45, 34%) and posterior trunk (44, 33%), (Table 3). Apart from the skin rash, old aged patients had cutaneous oedema around the ankles (Figure 4) and puffiness of face with the skin becoming dusky discolouration. Sixteen (12%) patients had pain and swelling of ankle joints where mild oedema extended to feet and mid leg suggestive of acute arthritis.

**Table 2:** Description of the skin rash: Category, colour, size and distribution.

Description	Number of patients; n,134 (%)
**Category of rash**	
**Maculopapular**	132 (98)
**Macular**	1 (1)
**Papular**	1 (1)
**Colour of rash**	
**Erythematous**	126 (94)
**Fern leaf type necrosis**	8 (6)
**Size of lesions- mm (mean, SD)**	5 (2)
**Extent of the distribution of rash; % (range)**	52 (13–100)

**Table 3:** Distribution of skin rash in the body.

Region^a^	Number of patients; n, 134 (%)
**Arms and forearms**	108(81)
**Legs**	90 (67)
**Soles**	75 (56)
**Palms**	74(55)
**Anterior body**	61(46)
**Thighs and gluteal regions**	53(40)
**Posterior trunk**	44 (33)
**Head and neck**	45 (34)

a Listed in the descending order of the frequency.

**Figure 5:**
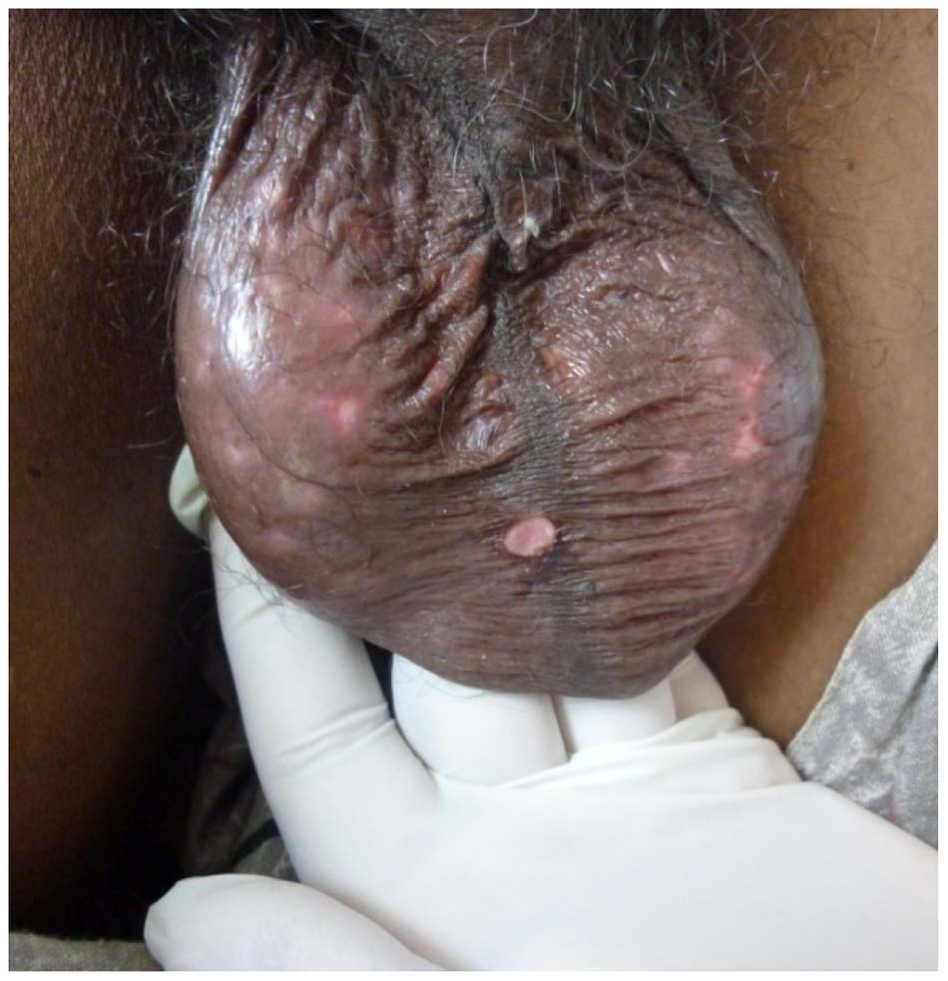
Scrotal swelling and gangrene of scrotal skin.

**Figure 6:**
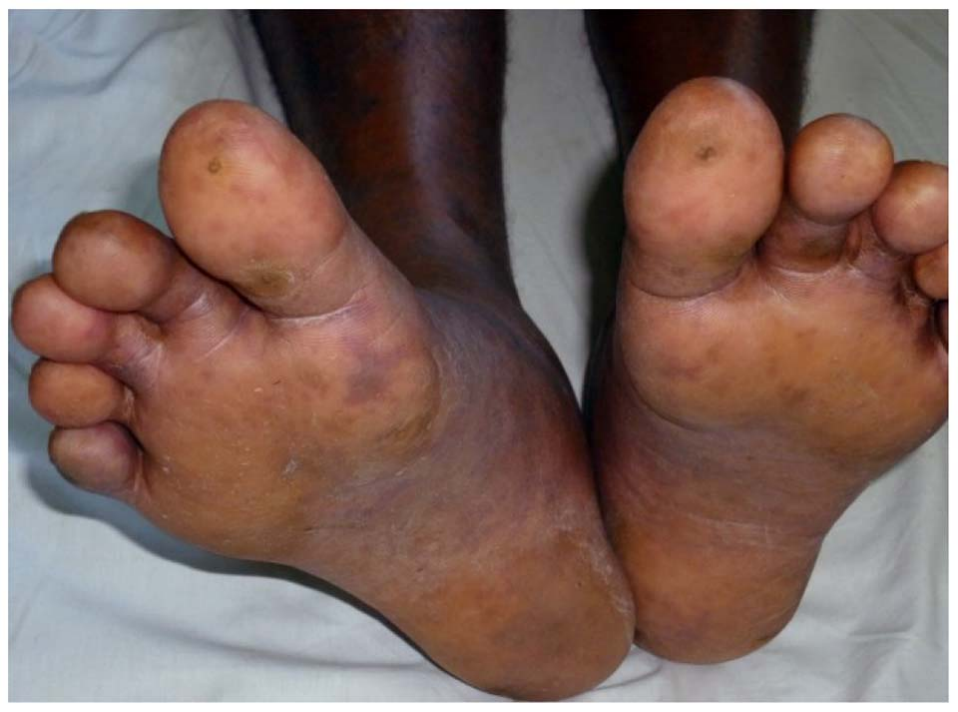
Vasculitic rash in toes and plantar surface of feet.

In 2012, a 65-year-old man presented with fever of 7 days and erythematous macular rash which progressed to patches of skin necrosis and gangrene of fern leaf shape mainly involving limbs. He had scrotal swelling and gangrene of scrotal skin (Figure 5). During the same period, a 64-year-old man presented with the similar history and developed dark brown skin lesions and vasculitic rash in toes and plantar surface of the feet (Figure 6). Both patients had positive titre of IFA for *Rickettsia conorii* antigens. They made recovery with doxycycline.

**Figure 7:**
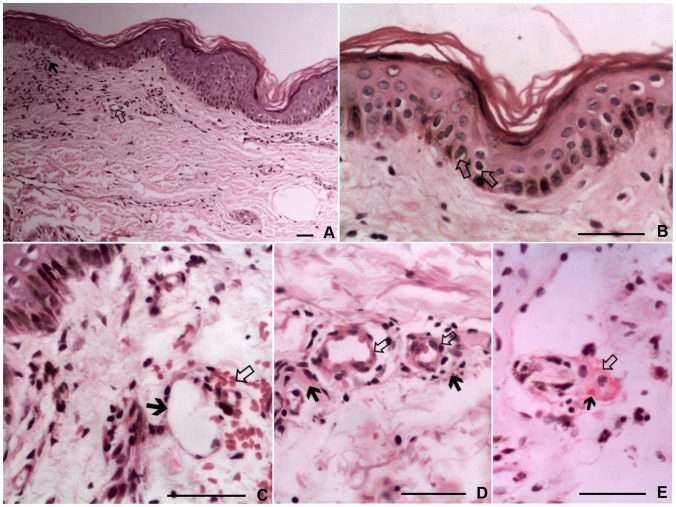
Cutaneous histopathology of maculopapular skin rash, (A) *Black arrow-* Exocytosis of extravasated lymphocytes. *White arrow* – Focal swelling of endothelium (haematoxyline-eosin; original magnification ×200), (B) Arrows indicate the vacuolations in the basal cells of epidermis, (C) *Black arrow* – Ectatic upper dermal blood vessels. *White arrow* – extravasated red blood cells, (D) *Black arrows* – Lymphocytic infiltrates in perivascular spaces. *White arrows* – Focal swelling of endothelium and ectatic upper dermal blood vessels, (E) *White arrow* – Fibrinoid necrosis of a dermal vessel wall. *Black arrow* – Presence of fibrin thrombi. (hematoxyline-eosin; original magnification ×800). Scale bars = 50 µm.

All six skin biopsy samples examined for histopathological characteristics showed evidence of cutaneous vasculitis. Main histopathological features were foci of basal cell vacuolation with exocytosis and lymphocytic infiltrates in perivascular spaces. Further, there were ectatic upper dermal blood vessels, focal swelling of endothelium, fibrinoid necrosis of vessel walls, extravasated red cells and presence of fibrin thrombi (Figure 7). The 247 bp region of the 17-kDa spotted fever group specific protein antigen was successfully amplified using PCR further confirming the presence of spotted fever among those patients.

## Discussion

This study describes both external morphology and histopathological features of cutaneous manifestations of spotted fever rickettsial infections in Sri Lanka where the causative rickettsial pathogens are yet unknown. All cases in the study group qualified for the diagnosis of spotted fever group rickettsioses, as the seroreactivity was noted only against *R. conorii* antigen, except in one patient who was positive both SFG and scrub typhus. Serological evidence with high antibody titres in acute serum samples alone has been considered confirmative based on previous studies [2], [3], [13]. However, rising titre in convalescent sera would have been the best. Due to practical difficulties, majority of patients failed to visit the clinic to give convalescent blood sample in two weeks after being discharged from the wards. Convalescent serum samples were tested only in six patients and all six showed a fourfold rise in antibody titre for SFG. The average duration between the acute and convalescent serum sample collection was two weeks. Moreover, we were aware of the concerns about serology such as IgM positivity that indicates a recent infection and is not necessarily the acute cause of fever. Further it is often found not very early during the course of infection and could give false positive results in other infections such as Epstein Barr viral infection. Also IgG positivity needs IgM or any other specific disease manifestations for confirmation. We used clinical case definition and high titre serology to overcome these issues.

None of the patients in this study group had classical eschars and eight patients had only possible tick bite marks. Classic eschars with thick and dry necrotic tissue have been described in some SFG rickettsioses such as Mediterranian spotted fever (MSF), African tick bite fever and rickettsial pox [14], [15]. Predominance of maculopapular erythematous rash in SFG has been reported earlier and the fern leaf type skin necrosis has not often been described other than in few studies done in the same region in Sri Lanka [2], [4]. However, there are reports of gangrene of digits, ear lobes, scrotum, nose or limbs occurring in rickettsial infections such as RMSF [16], similar to two cases presented in 2012. Even though, the skin rash had a wide distribution in different regions of the body, majority had dominant involvement of arms, forearms and legs. Although the involvement of palms and soles are characteristic in rickettsial infections, only about 56% of this group had the rash involving palms and soles. Moreover, the study describes some important general characteristics basically seen in old aged patients. These include cutaneous oedema around the ankles and puffiness of face with the skin becoming dusky discolouration. Importantly, such focused descriptions are not found in earlier studies. Even though, majority of patients did not come for follow up, our experience suggests that cutaneous rashes take 2–3 weeks to fade off after defervescence. Furthermore, rash negative forms of rickettsial cases have also been described from Sri Lanka amounting to a smaller percentage [18].

Clinical features of SFG of Sri Lanka described in the current study do differ from the presenting features of SFG in some regions of the globe. RMSF is a well known SFG in America, caused by *R.rickettsii*, used to present with high fever, headache, myalgia and skin rash as the predominant clinical manifestations. However eschars are rare in this disease and skin necrosis has been reported [17], [19]. Generally the maculopapular skin rash is mainly distributed in the limbs. Commonest SFG rickettsioses reported in Southern Europe and Northern Africa is MSF caused by *R.conorii*. In contrast to SFG in Sri Lanka and RMSF, eschars are common in MSF where the distribution of maculopapular or petichial skin rash in palms and soles is remarkable. In addition to these, R. *japonica* causes Japanese spotted fever mainly in East Asia. Major clinical manifestations of Japanese spotted fever are headache, fever, skin rash and eschars [1]. There are about 18 different SFG agents identified from different regions of the globe with variations in clinical picture and mode of transmission. This vast diversity can mainly be attributed to geographical factors, socio-economic factors and vector-human relationships [1], [14]. The cutaneous histopathology of SFG rickettsioses is caused by endothelial damage by the rickettsial organisms. As per the previous findings the basic histopathological changes that can be seen in skin eruptions and patchy necrotic lesions include lymphohistiocytic capillaritis and venulitis, perivascular oedema, erythrocyte extravasation, interstitial infiltrate, leukocytoclastic vasculitis and presence of nuclear dust. There can also be epidermal changes including basal layer vacuolar degeneration with mild dermoepidermal interface lymphocytic exocytosis, focal fibrin thrombi, capillary wall necrosis and perivascular inflammation [10]. Most of these were evident in this study and besides there were small vessel ectasis and focal swelling of endothelium which are also supportive of the vasculitis. This is the first time a study described histopathological features of the cutaneous lesions of rickettsioses in Sri Lanka. Furthermore, use of immunohistochemical stains may have identified the causative rickettsial organisms in the skin lesions.

### Conclusions

Pattern of cutaneous manifestations have a pivotal role in making a clinical diagnosis of SFG. The typical features of the skin rash include discrete maculopapular lesions with dusky erythemtous hue, distributed mainly in the limbs, back of the chest, anterior abdomen and soles. Variations are common, such as fern leaf pattern of skin necrosis mainly involving superficial skin with blackish discoloration which with time dries up and peels off. Mild cutaneous oedema is common over the ankles and face specially in older patients. Histological features like disruption of small vessels by inflammatory cells, deposition of fibrin thrombi within the lumen and leukocytic infiltrates in perivascular spaces suggest vasculitis caused by infecting organism.

## Supporting Information

Checklist S1 STROBE checklist.
(DOC)

## References

[1] Parola P, Paddock CD, Socolovschi C, Labruna MB, Mediannikov O, et al (2013) Update on tick-borne rickettsioses around the world: a geographic approach. Clin Microbiol Rev 26 (4) 657–702. 24092850 10.1128/CMR.00032-13 PMC3811236

[2] Kularatne SA, Edirisingha JS, Gawarammana IB, Urakami H, Chenchittikul M, et al (2003) Emerging rickettsial infections in Sri Lanka: the pattern in the hilly Central Province. Trop Med Int Health 8 (6) 803–811. 12950666 10.1046/j.1365-3156.2003.01108.x

[3] Premaratna R, Loftis AD, Chandrasena TG, Dasch GA, de Silva HJ (2008) Rickettsial infections and their clinical presentations in the Western Province of Sri Lanka: a hospital-based study. Int J Infect Dis 12 (2) 198–202. 17900956 10.1016/j.ijid.2007.06.009

[4] Kularatne SAM, Rajapakse RPVJ, Wickramasinghe WMRS, Nanayakkara DM, Budagoda SS, et al (2013) Rickettsioses in the central hills of Sri Lanka: serological evidence of increasing burden of spotted fever group. Int J Infect Dis 17 (11) e988–992. 23871280 10.1016/j.ijid.2013.05.014

[5] Walker DH (1995) Rocky Mountain spotted fever: A seasonal alert. Clin Infect Dis 20: 1111–1117. 7619984 10.1093/clinids/20.5.1111

[6] Raoult D, Parola P (2008) Rocky Mountain spotted fever in the USA: a benign disease or a common diagnostic error. Lancet Infect Dis 8 (10) 587–589. 18922479 10.1016/S1473-3099(08)70210-X

[7] Parola P, Raoult D (2006) Tropical rickettsioses. Clin Dermatol 24 (3) 191–200. 16714200 10.1016/j.clindermatol.2005.11.007

[8] Saxena A, Khiangte B, Tiewsoh I (2014) Scrub typhus complicated by acute respiratory distress syndrome and multiorgan failure; an unrecognized alarming entity in central India: a report of two cases. J Family Med Prim Care 3 (1) 80–83. 24791245 10.4103/2249-4863.130334 PMC4005210

[9] Amaro M, Bacellar F, França A (2003) Report of eight cases of fatal and severe Mediterranean spotted fever in Portugal. Ann N Y Acad Sci 990: 331–343. 12860647 10.1111/j.1749-6632.2003.tb07384.x

[10] Kao GF, Evancho CD, Ioffe O, Lowitt MH, Dumler JS (1997) Cutaneous histopathology of Rocky Mountain spotted fever. J Cutan Pathol 24 (10) 604–610. 9449487 10.1111/j.1600-0560.1997.tb01091.x

[11] Liyanapathirana VC, Thevanesam V (2011) Seroepidemiology of rickettsioses in Sri Lanka: a patient based study. BMC Infect Dis 11 (1) 328. 22118601 10.1186/1471-2334-11-328 PMC3248378

[12] Premaratna R, Weerasinghe S, Ranaweera A, Chandrasena TN, Bandara NW, et al (2012) Clinically helpful rickettsial disease diagnostic IgG titers in relation to duration of illness in an endemic setting in Sri Lanka. BMC Res Notes 30 (5) 662. 10.1186/1756-0500-5-662 PMC3536648 23198969

[13] Premaratna R, Rajapakse RP, Chandrasena TG, Nanayakkara DM, Bandara NK, et al (2010) Contribution of rickettsioses in Sri Lankan patients with fever who responded to empirical doxycycline treatment. Trans R Soc Trop Med Hyg 104 (5) 368–370. 19931108 10.1016/j.trstmh.2009.10.006

[14] Parola P, Paddock C, Raoult D (2005) Tick-borne rickettsioses around the world: emerging diseases challenging old concepts. Clin Microbiol Rev 18: 719–756. 16223955 10.1128/CMR.18.4.719-756.2005 PMC1265907

[15] Richter J, Fournier PE, Petridou J, Hausinger D, Raoult D (2002) Rickettsia felis infection acquired in Europe and documented by polymerase chain reaction. Emerg Infect Dis 8: 207–208. 11897076 10.3201/eid0802.010293 PMC2732449

[16] McBride WJ, Hanson JP, Miller R, Wenck D (2007) Severe spotted fever group rickettsiosis, Australia. Emerg Infect Dis 13 (11) 1742–1744. 18217560 10.3201/eid1311.070099 PMC3375793

[17] Kirkland KB, Marcom PK, Sexton DJ, Dumler JS, Walker DH (1993) Rocky Mountain spotted fever complicated by gangrene: report of six cases and review. Clin Infect Dis 16 (5) 629–634. 8507753 10.1093/clind/16.5.629

[18] Reller ME, Bodinayake C, Nagahawatte A, Devasiri V, Kodikara-Arachichi W, et al (2012) Unsuspected rickettsioses among patients with acute febrile illness, Sri Lanka. Emerg Infect Dis 18 (5) 825–829. 22516455 10.3201/eid1805.111563 PMC3358078

[19] Griffith GL, Luce EA (1978) Massive skin necrosis in Rocky Mountain spotted fever. South Med J 71 (11) 1337–1340. 362535 10.1097/00007611-197811000-00007

